# National policies for infertility support and nursing strategies for patients affected by infertility in South Korea

**DOI:** 10.4069/kjwhn.2021.03.12.1

**Published:** 2021-03-26

**Authors:** Miok Kim

**Affiliations:** Department of Nursing, College of Nursing, Dankook University, Cheonan, Korea

In December 2020, the Korean government announced the 2021–2025 Fourth Basic Plan for Low Fertility and Aged Society [1]. Reflecting social and environmental changes, the fourth plan includes a comprehensive lifelong guarantee of sexual and reproductive health rights. The plan supports healthy pregnancy and childbirth by strengthening social support for healthy and safe contraception, maintenance and termination of pregnancy, expanded resources for high-risk pregnancies, and safe infertility care centered around consumers. The *Issues and Perspectives* [2] published in this issue highlights the background of the plan and reviews the phenomenon of the low birth rate in detail. In particular, a suggestion is made to improve the safety of infertility treatments for the health of mothers and children and to provide psychological/emotional support such as information provision and counseling. In this article, I would like to summarize the present state of infertility support, including financial support for infertility treatment and psychological/emotional support such as information provision and counseling, as well as sharing my thoughts on the relevant arrangements.

## Korea’s infertility support policy

The number of people diagnosed with infertility in Korea increased from 185,000 in 2010 to 200,000 in 2014 [3], and further grew to over 230,000 in 2019 [4]. Against this backdrop, the government initiated a project to provide financial support for infertility treatment in 2006, thereby standardizing infertility treatment procedures that were not previously covered by insurance. Health insurance coverage for infertility treatment procedures was subsequently applied from October 2017. Accordingly, from October 2017 to September 2018, approximately 120,000 patients benefitted from health coverage for infertility treatment, to the tune of 1.2 million USD (based on the amount reviewed and determined by the Health Insurance Review and Assessment Service excluding duplicate patients) [5].

In response to strong public advocacy supporting the expansion of health insurance coverage to include infertility treatment, the government abolished the restriction on the age of women to 44 years or younger (international age) for insurance-covered infertility treatment in July 2019, and expanded coverage so that women aged 45 years or older (international age) could benefit from health insurance for infertility treatment if considered necessary based on the medical assessment of a physician. Furthermore, the number of in vitro fertilization (IVF) and intrauterine insemination (IUI) procedures covered by health insurance was increased to a limited extent (by increasing the co-pay for additional procedures from 30% to 50%) and efforts to relieve women’s economic and psychological burden were made by establishing infertility/depression counseling centers.

In other words, in 2019, health insurance coverage for fertility-related counseling/education and examinations was promoted to encourage couples thinking of having children to actively monitor their physical condition before being diagnosed as infertile and to prevent infertility in advance. In addition, the government developed a plan to apply health insurance coverage to basic infertility tests at medical institutions, steps taken to achieve an appropriate physical condition for pregnancy, and education and counseling on conception for any couples planning to conceive a child, as well as a comprehensive plan to create a safe, healthy delivery environment for couples struggling with infertility and to address social needs.

The government is striving to improve the standard for the number of transplanted embryos, devise a plan to improve the safety of procedures, provide information necessary before and after infertility procedures on the public information portal, strengthen psychoemotional support such as counseling for infertile couples, offer health information on women who underwent infertility treatment and their babies, provide information on the status of use of infertility/depression counseling centers, and present evaluations of satisfaction on the comprehensive parenting portal. Furthermore, steps are being taken to support childcare and extended leave for infertility treatment, all of which are measured aimed at strengthening consumer-centered safe infertility support.

## Provision of infertility-related information and psychological/emotional support

The emotional difficulties experienced by women during infertility treatment, combined with experiences of pain, discomfort, and physical fatigue during assisted reproductive procedures such as ovarian stimulation and invasive ovum aspiration, may contribute to low infertility treatment success rates, reduced quality of life [6,7], and decisions to discontinue treatment [8].

In light of the recognition that psychosocial support improves both the likelihood of conception and the quality of life of people experiencing infertility, thereby ultimately improving the effectiveness of infertility support, major advanced countries have assigned and managed infertility counseling as a main component of infertility procedures and treatment. For example, in the United Kingdom, Australia, and New Zealand, infertility counseling has been mandated through legislation so that prior to infertility treatment, sufficient general information on infertility and infertility-related procedures are sufficiently provided to aid in the decision-making process. In addition to providing medical information on infertility and supporting the decision-making process, these countries have expanded the scope of infertility counseling to a wide range of psychological health issues, including the psychological distress of infertile women, relationship problems between partners and with family members, and personal quality of life and satisfaction [9]. These psychological interventions have been reported to produce positive results in terms of relieving depression in women undergoing infertility treatment and improving marital intimacy, sexual satisfaction, fatigue [10], and pregnancy rates [11].

The provision of information and psychological support for infertile couples in Korea was mainly conducted at medical institutions providing infertility treatment in the past. However, the Korea Population, Health and Welfare Association conducted a pilot operation of an infertility counseling center in 2015 and opened the Central Infertility and Depression Counseling Center in June 2018. Subsequently, additional centers were opened in the Jeonnam, Incheon, and Daegu areas to provide information on mental health and improve emotional and psychological health, as well as supporting medical procedures related to infertility, pregnancy, and childbirth. As a result, statistically significant relief in depression, anxiety, and stress has been reported in women who received services at the infertility/depression counseling center [12].

## Infertility nursing strategies

Women who receive infertility treatment experience both physical and psychological fatigue throughout the treatment process [13]. Physical fatigue due to frequent hospital visits and invasive procedures for the diagnosis and treatment of infertility may impose a burden on women receiving infertility treatment in combination with psychological difficulties and tension during ovulation induction, which is performed to assess the growth rate and number of follicles, as well as anxiety and tension during procedures such as IUI and IVF, and psychological fatigue as pregnancy is confirmed [13].

Women who undergo assisted reproductive technology treatment have higher levels of negative emotions before IVF than during IVF [14], and couples who undergo repeated cycles have higher levels of depression than those who have just started IVF for the first time [15]. In addition, the risk of depression is high until 1 year after successful IVF and childbirth [16], and women who experienced IVF in the past were found to have a higher risk of depression 20 to 23 years later regardless of childbirth [17]. The quality of life of women undergoing IVF is lower when their level of depression is high, previous treatment has failed, and the cause of infertility is unknown; the latter two factors are particularly important contributors to the deterioration of quality of life [18]. Therefore, differentiated intervention strategies are required according to the cause of infertility, and more psychoemotional interventions are needed when infertility treatment fails.

When positive interactions do not occur in a marital relationship, stress and negative emotions related to infertility are directly transmitted without filtering, resulting in a vicious cycle of negative emotions involving conflict, regret, and feelings of guilt [13]. Such psychological discomfort and difficulties associated with infertility can be alleviated if women receiving fertility treatment understand their own emotions and express them appropriately through words and actions [13]. Indirect exposure of women undergoing infertility treatment to media has a negative correlation with infertility-related quality of life, whereas a positive association with overall quality of life has been demonstrated for the direct and gradual disclosure of methods that provide opportunities for face-to-face meetings, verbal expression, and immediate responses [19]. Therefore, women who undergo infertility treatment should be sensitive to their feelings and changes due to infertility, and at the same time, they should be able to express their feelings appropriately, for which interventions that help effective communication and positive interactions with their spouses are required.

In the meantime, a high level of stigma perceived by women is related to a low quality of life associated with infertility. In light of the fact that 88.1% of patients receiving infertility treatment need psychological counseling [20], it is necessary to improve perceptions of infertility in individuals and in society, along with providing psychological support [21], in order to reduce the stigma of infertility and improve quality of life.

Numerous studies have reported physical, psychoemotional, and socioeconomic difficulties among women diagnosed with infertility and receiving treatment in Korea. These difficulties are similar for women with female factor infertility and for those with unknown causes of infertility [13], and the burden of male factor infertility is also imposed on women [22]. Infertility should be understood as a situational difficulty that requires more effort than other challenges because of the possible or actual presence of factors that make it difficult to succeed in pregnancy, rather than being understood as a woman's individual defect or insufficiency. It would also be desirable to use the terms “women receiving infertility treatment” and “women receiving IVF” instead of “infertile women,” which has the effect of stigmatizing them. Furthermore, the main strategy for overcoming infertility could be to make ongoing efforts to positively change the perceptions of women who are diagnosed with infertility and those of Korean society toward infertility.

With a multifaceted policy that helps alleviate socioeconomic difficulties accompanying the process of diagnosis and treatment of infertility, medical institutions that provide infertility treatments and local communities should establish strategies to reduce physical difficulties for women receiving infertility treatment and to improve psychoemotional well-being at the personal and interpersonal levels throughout the process of planning infertility treatment, failure, and further attempts. In other words, it is necessary to examine patients’ psychoemotional status, as well as physical difficulties, experienced during the infertility treatment process and to provide counseling accordingly. Couples should also be included as recipients of infertility nursing care throughout the process of infertility treatment; they should be provided sufficient information, and it is necessary to develop and apply multifaceted interventions at the individual, family, and society levels to help people overcome infertility together with active support from their spouses.
